# Supplementary material for: Spectroscopic studies of atomic defects and bandgap renormalization in semiconducting monolayer transition metal dichalcogenides
Source: Nat Commun. 2019 Aug 23;10:3825. doi: 10.1038/s41467-019-11751-3 (PMC6707146; doi:10.1038/s41467-019-11751-3)
Supplement: Supplementary file 1 — Supplementary Information [file 41467_2019_11751_MOESM1_ESM.pdf]

Supplementary Information

Spectroscopic studies of atomic defects and bandgap renormalization in semiconducting monolayer transition metal dichalcogenides

Jeong et al.

Supplementary Figures

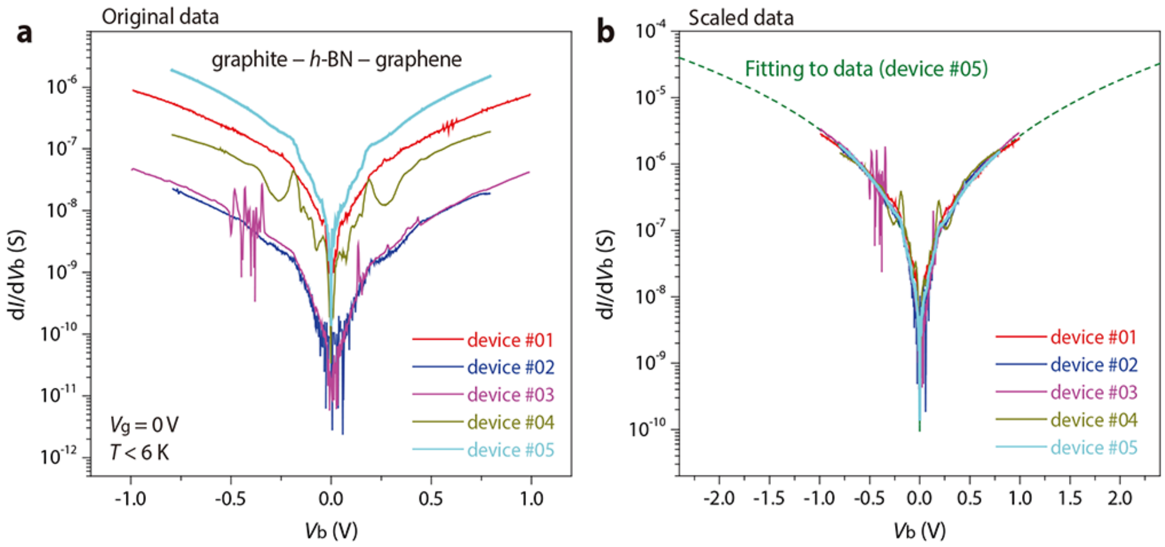

Supplementary Figure 1. Electron tunneling spectra of graphite–*h*-BN–graphene tunnel junctions. **a**, Series of dI/dV_b spectra from multiple planar tunnel junctions of single-layer graphene with graphite as a probe and thin *h*-BN as a tunnel barrier. Dirac points for all graphene tunnel junctions are close to the Fermi level (V_b = 0 mV) at V_g = 0 V. V_b is applied to the graphite probe and tunnel current is monitored through the graphene layer, in an identical setup as the measurements in the main text. Differences in dI/dV_b values are ascribed to tunnel *h*-BN layers and tunnel junction area. **b**, Scaled tunneling spectra adjusted with dI/dV_b multiplication constants to overlay with the dI/dV_b of device #05 (solid cyan line). Dotted green lines represent numerical fittings to the experimental data of device #05, requiring different fitting parameters for positive and negative V_b regions, which exhibit the electron–hole asymmetric electronic structures of the graphite–*h*-BN–graphene planar tunnel junctions. This fitting data, referred to as the graphene baseline, is used to analyze the electron tunneling spectra of the SC-mTMD-based planar junctions in the main text.

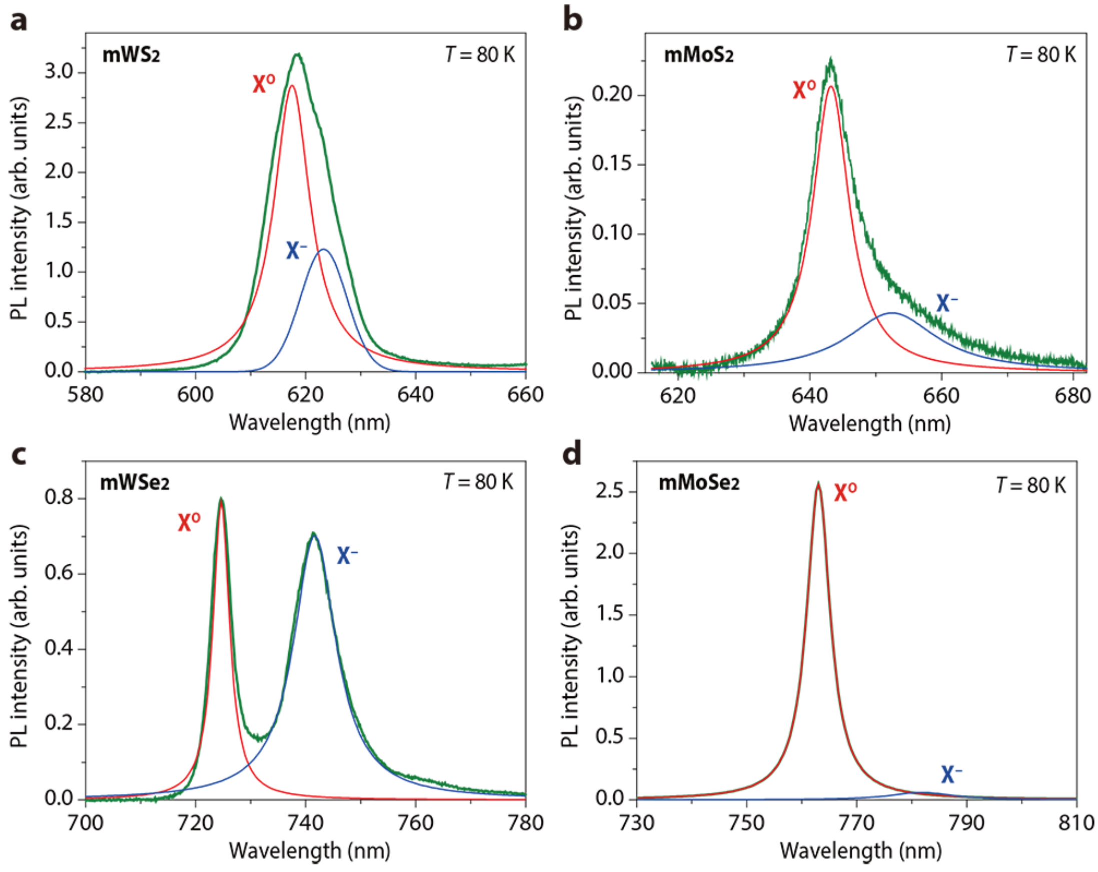

Supplementary Figure 2. Photoluminescence (PL) measurements of the SC-mTMD films. PL spectra displaying *A*-exciton peaks of the mWS_2 (**a**), $mMoS_2$ (**b**), $mWSe_2$ (**c**), and $mMoSe_2$ (**d**) films at T = 80 K. PL signals are collected right at the junction where the respective films are encapsulated by *h*-BN, graphite, and graphene layers in the planar tunnel junction platform. Pump light, CW Nd:YAG laser centered at 532 nm, is vertically shone onto the flakes through a ×50 objective lens, and PL signals are collected through the same objective lens and analyzed with a liquid-nitrogen cooled Si CCD detector with 550 nm long pass filter. The positions of the *A*-exciton (X^O) peaks are determined by Lorentzian fittings with consideration of nearby charged excitons (X^-).

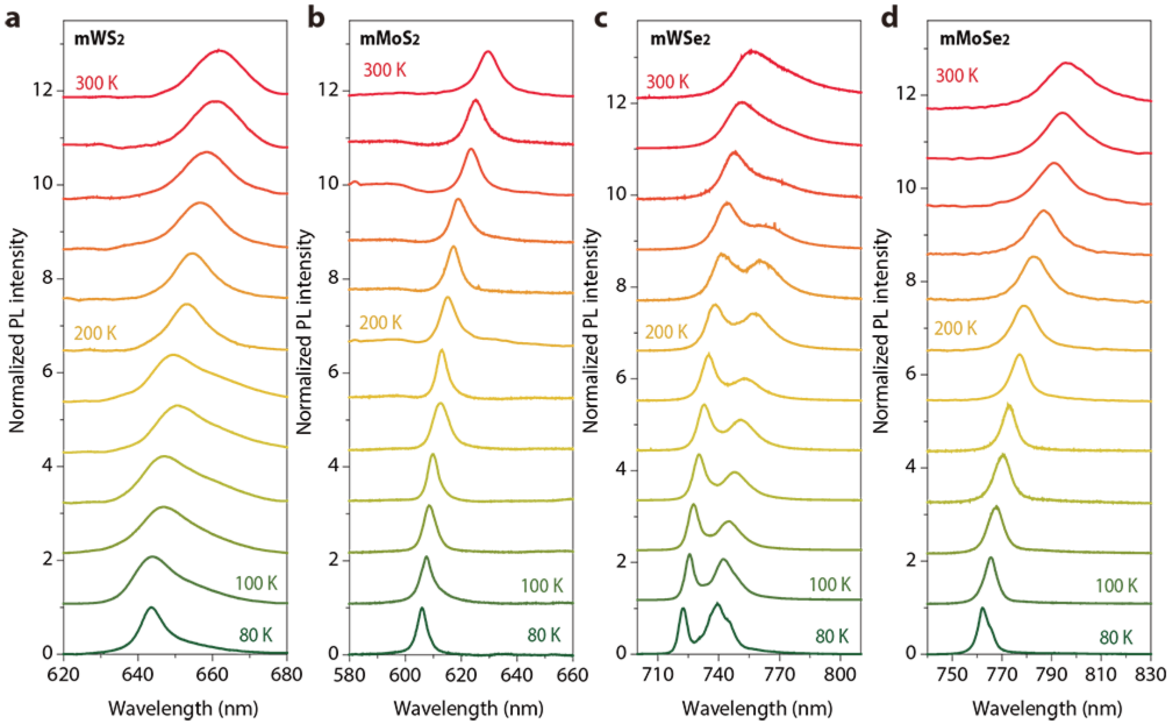

Supplementary Figure 3. Temperature-dependent PL measurements of the SC-mTMD films. Normalized PL spectra of mWS_2 (**a**), $mMoS_2$ (**b**), $mWSe_2$ (**c**), and $mMoSe_2$ (**d**) measured in the temperature range 80 K $\leq T \leq$ 300 K. Each spectrum is normalized to the maximum PL intensity. Moderate blue-shifts of the *A*-exciton peaks are universal in all four films.

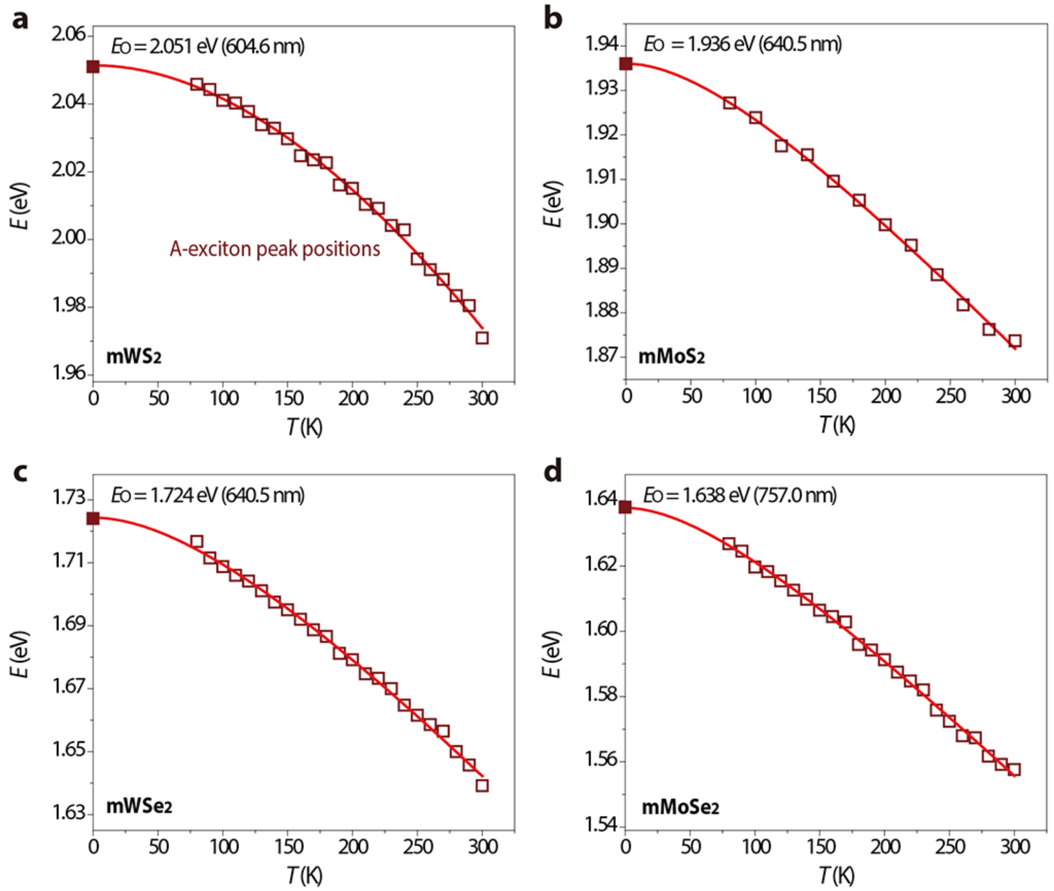

Supplementary Figure 4. Extracting the optical energy gaps of the SC-mTMD films. a–d Series of *A*-exciton peaks of the respective SC-mTMD films measured in the temperature range 80 K ≤ *T* ≤ 300 K. Continuous blue-shift of the *A*-exciton peaks with decreasing temperature allows for the extraction of the intrinsic optical energy gaps of the films by following the peak positions to *T* = 0 K, utilizing the Varshni relation that explains the temperature dependence of semiconductor energy gaps:

$$E_g(T) = E_o - \frac{\alpha T^2}{T + \beta},$$

where E_o is the energy gap at $T = 0$ K and α and β are material-dependent fitting parameters.

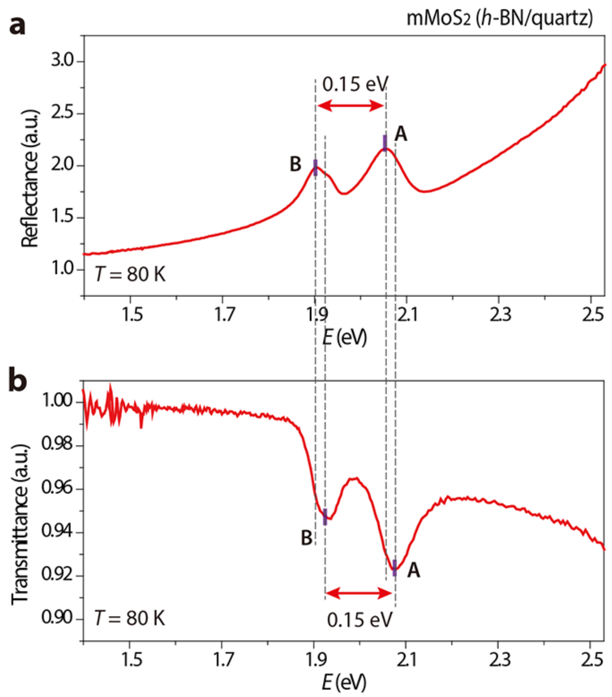

Supplementary Figure 5. Optical reflectance and transmittance measurements of $mMoS_2$. Optical reflectance (**a**) and transmittance (**b**) spectra of $mMoS_2$ film at T = 80 K. The film is transferred onto thick *h*-BN layers and then placed on a transparent quartz substrate. For reflectance and transmittance measurements, supercontinuum white source (NKT SuperK COMPACT supercontinuum lasers) is vertically shone onto the $mMoS_2$ film, mounted in a liquid nitrogen-cooled cryostat. Both optical measurements clearly show signals relating to the *A* and *B* excitons of the $mMoS_2$ film. The *A*–*B* exciton spacing, thereby spin-orbit coupling (SOC) induced valence band splitting of the $mMoS_2$, is estimated to be Δ_{SO} ($mMoS_2$) = 0.15 ± 0.01 eV. Note that individual exciton peak (**a**) and dip (**b**) positions in the reflectance and transmittance optical spectra are slightly off, attributed to different physical mechanisms in optical measurements.

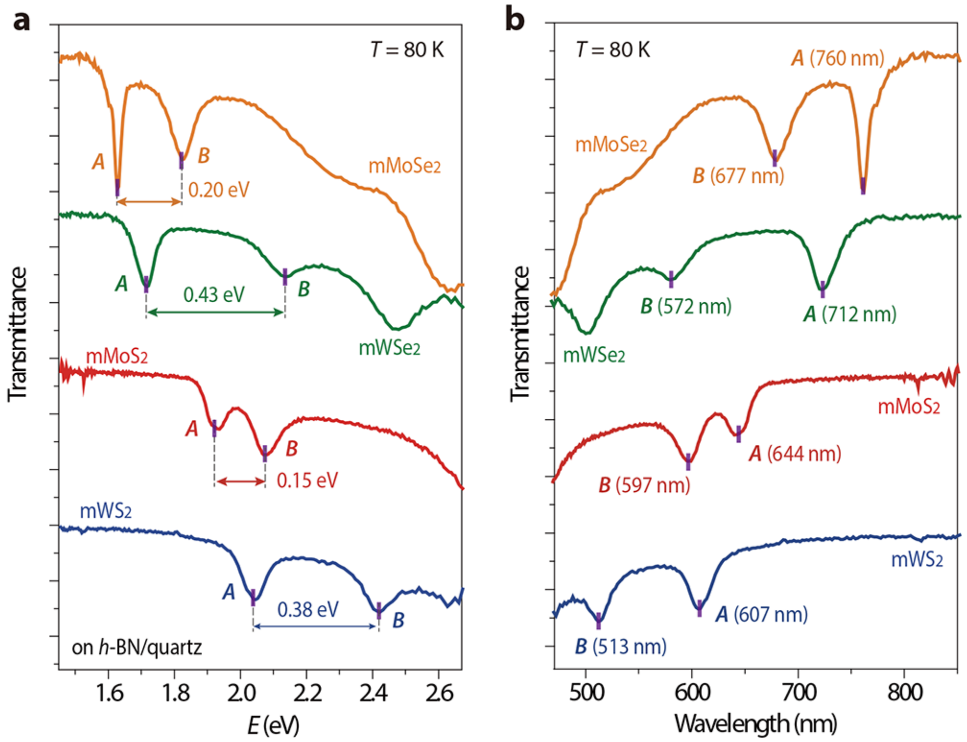

Supplementary Figure 6. *A*–*B* exciton spacing in SC-mTMD films. Series of optical transmittance spectra of the four respective films at T = 80 K, fabricated onto *h*-BN layers and a transparent quartz substrate at varying energy (**a**) and wavelength (**b**). The clearly resolved *A*–*B* excitonic peak spacings allow us to determine the SOC-induced valence-band splittings in all four films at high accuracy with an uncertainty level of less than ± 0.01 eV. Individual peak positions are extracted from numerical fittings to Lorentzian functions with baseline subtractions.

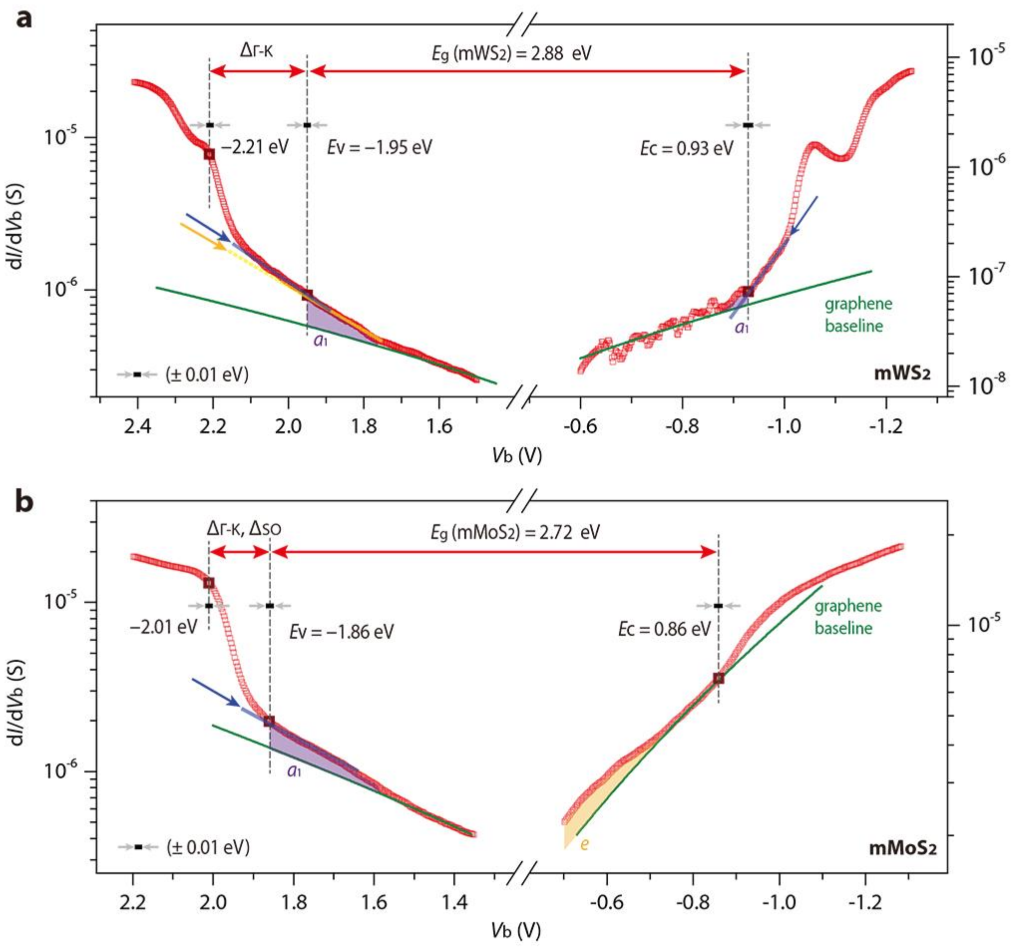

Supplementary Figure 7. Detailed electronic structure analyses of mWS_2 and $mMoS_2$ films. dI/dV_b tunneling spectra focused around the valence- and conduction-band edges of S-based mTMD films mWS_2 (**a**) and $mMoS_2$ (**b**). Valence band edges (the higher SOC-split band edge at the K point) are located based on the optically addressed *A*–*B* exciton spacings (Supplementary Figure 6) and eminent dI/dV_b features as marked with open brown squares. Conduction band edges are similarly located where the graphene baseline (green lines) and dI/dV_b spectra start deviating.

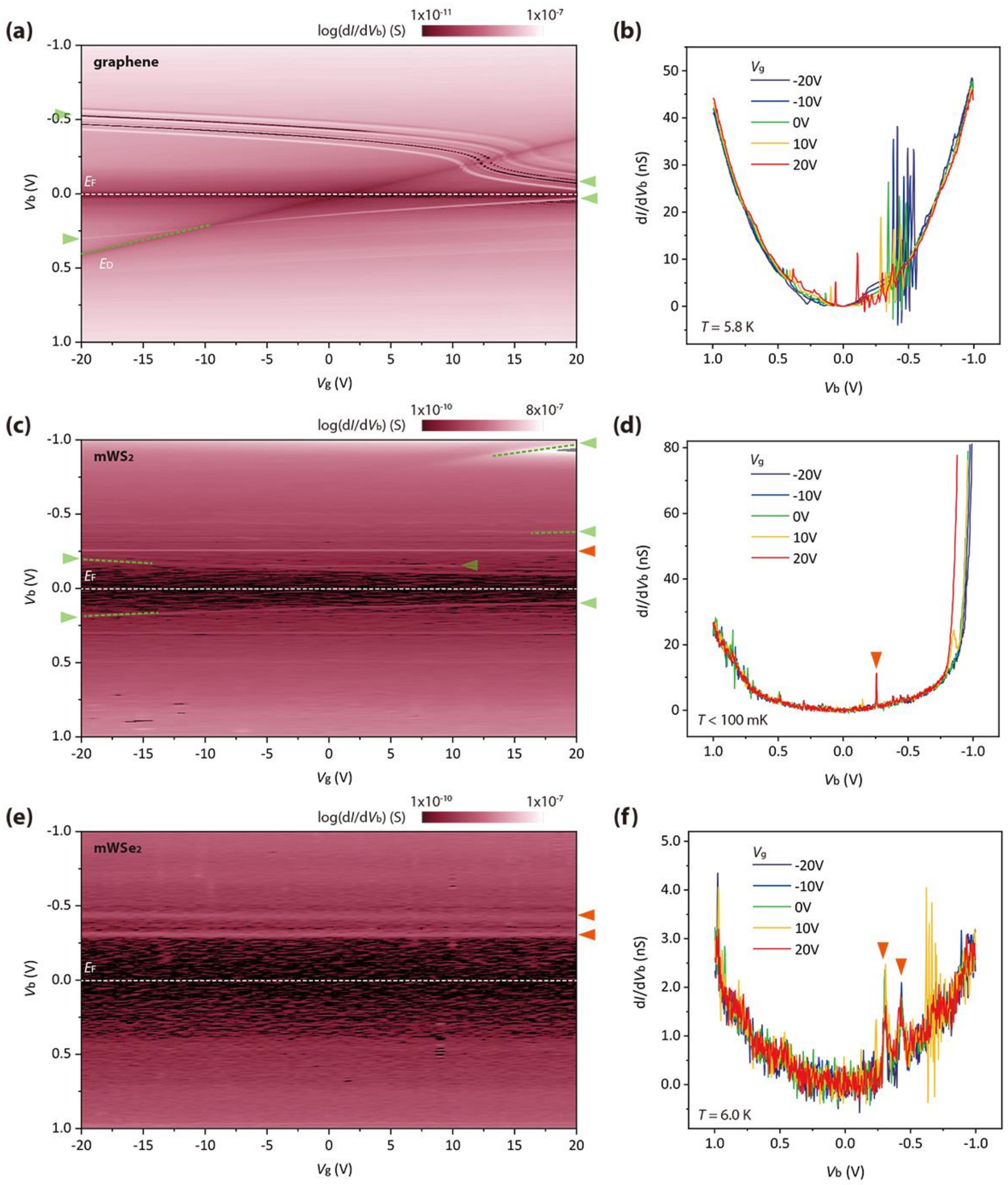

Supplementary Figure 8. Gate mappings and individual tunnel spectra for different tunnel junctions. Two-dimensional display of dI/dV_b curves at varying V_b and V_g for graphene (**a**), mWS_2 (**c**), mWSe_2 (**e**) based planar heterojunctions with a graphite as a tunnel probe and a thin *h*-BN as a tunnel insulator. The collections of individual dI/dV_b spectra at varying V_g are shown in (**b**) for the graphene, (**d**) for the mWS_2, and (**f**) for the mWSe_2 planar junctions.

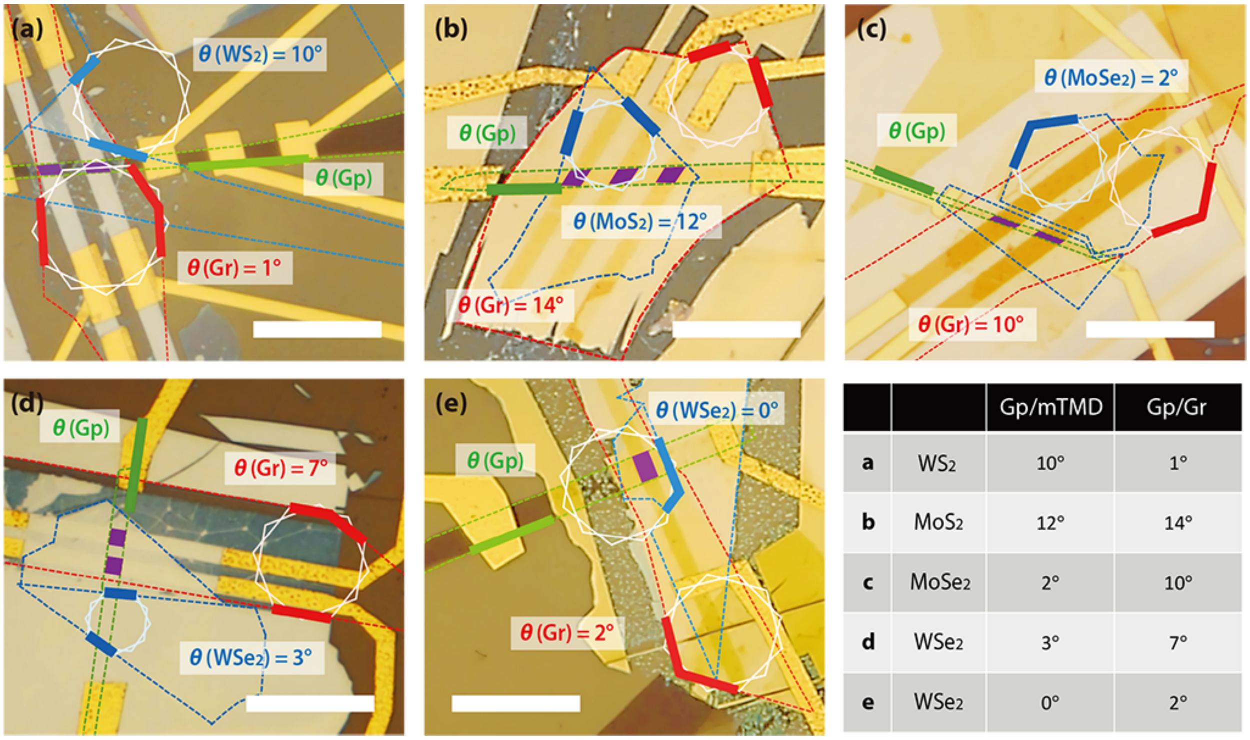

		Gp/mTMD	Gp/Gr
a	WS_2	10°	1°
b	MoS_2	12°	14°
c	$MoSe_2$	2°	10°
d	WSe_2	3°	7°
e	WSe_2	0°	2°

Supplementary Figure 9. Optical viewgraphs of SC-mTMD-based planar tunnel junctions. Optical images and the twist angle of SC-mTMD films and underlying graphene with respect to the top graphite for (**a**) mWS_2, (**b**) $mMoS_2$, (**c**) $mMoSe_2$, and $mWSe_2$ devices without (**d**) and with (**e**) angles aligned. Scale bars are 20 μm.

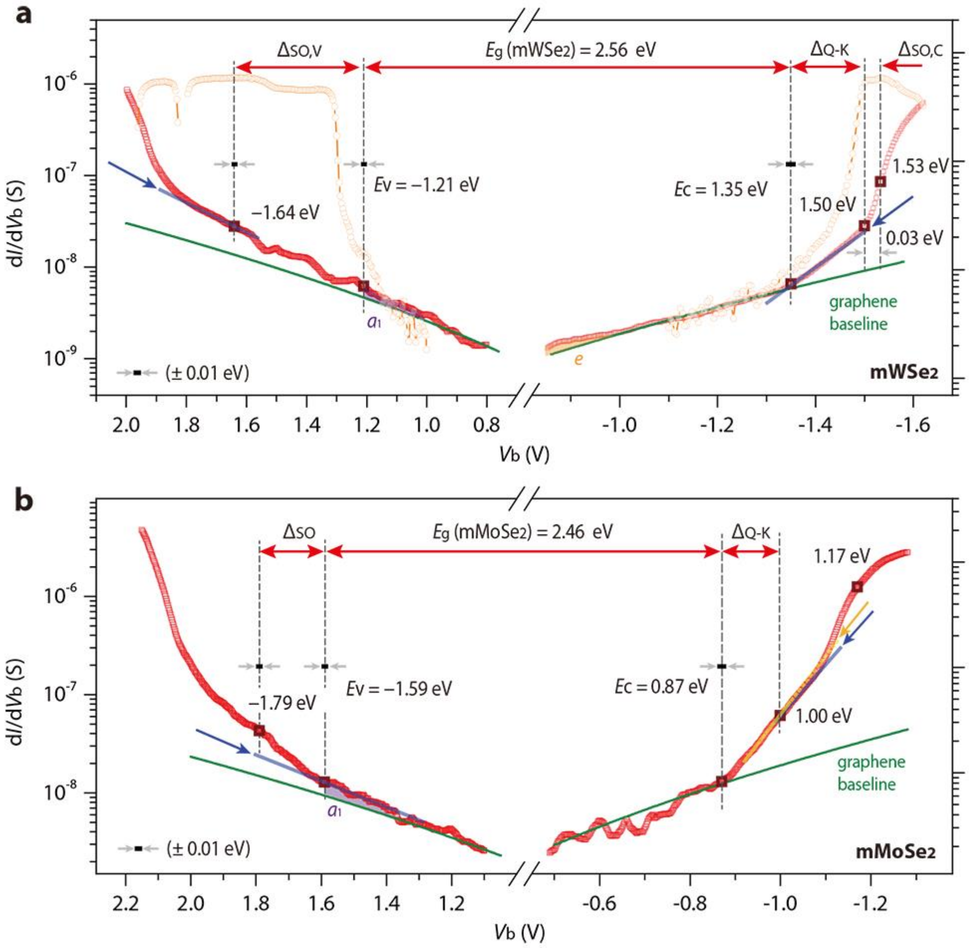

Supplementary Figure 10. Detailed electronic structure analyses of $mWSe_2$ and $mMoSe_2$ films. dI/dV_b tunneling spectra focused around the valence- and conduction-band edges of Se-based mTMD films $mWSe_2$ (**a**) and $mMoSe_2$ (**b**). Valence band edges and conduction band edges are found in the same manner as in Supplementary Figure 7.

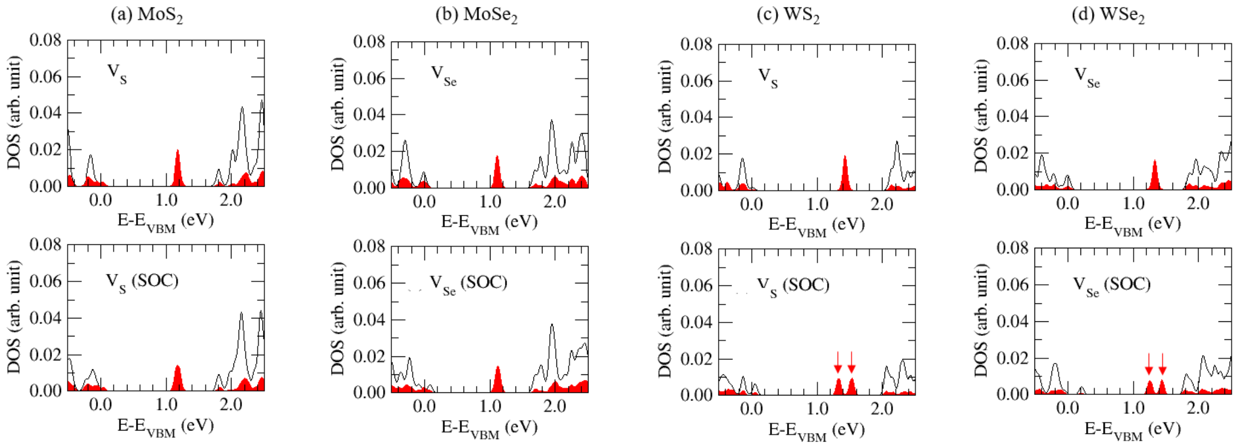

Supplementary Figure 11. DFT results on single chalcogen-atom vacancies. a–d, Calculated defect density of states induced from single chalcogen-atom vacancies (V_S or V_{Se}) in four SC-mTMD films of (**a**) MoS_2, (**b**) $MoSe_2$, (**c**) WS_2, and (**d**) WSe_2 without (upper row) and with (lower row) consideration of SOC effects. The splits of the doublet *e* defect states in WS_2 and WSe_2 with the SOC are marked with red arrows.

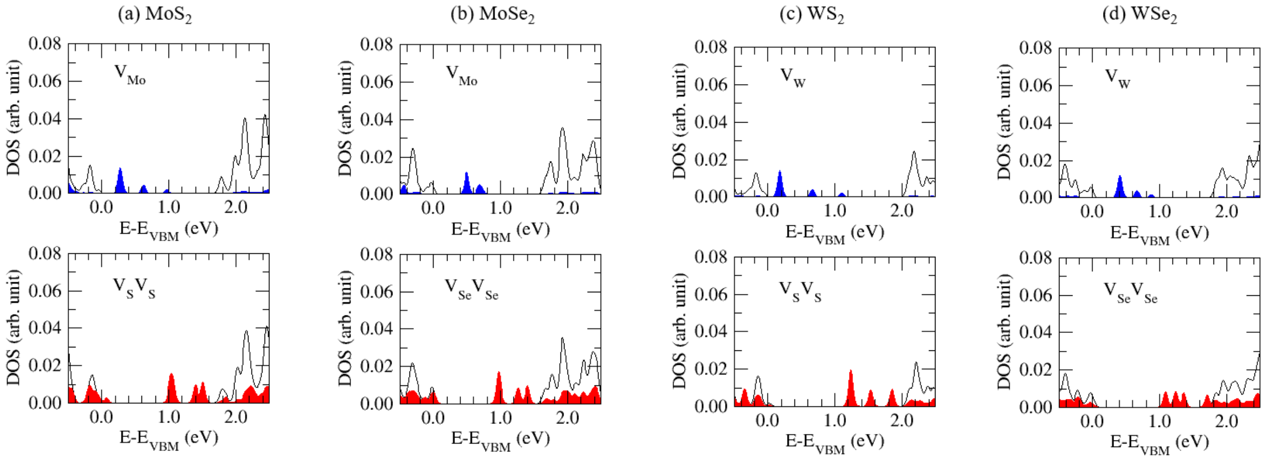

Supplementary Figure 12. DFT results on single transition metal-atom and double chalcogen-atom vacancies. a–d, Calculated defect DOS for four SC-mTMD films of (**a**) MoS_2, (**b**) $MoSe_2$, (**c**) WS_2, and (**d**) WSe_2 from the missing of single transition-metal atoms (upper row, V_{Mo} or V_W) and double chalcogen-atom vacancies (lower row, V_SV_S or $V_{Se}V_{Se}$).

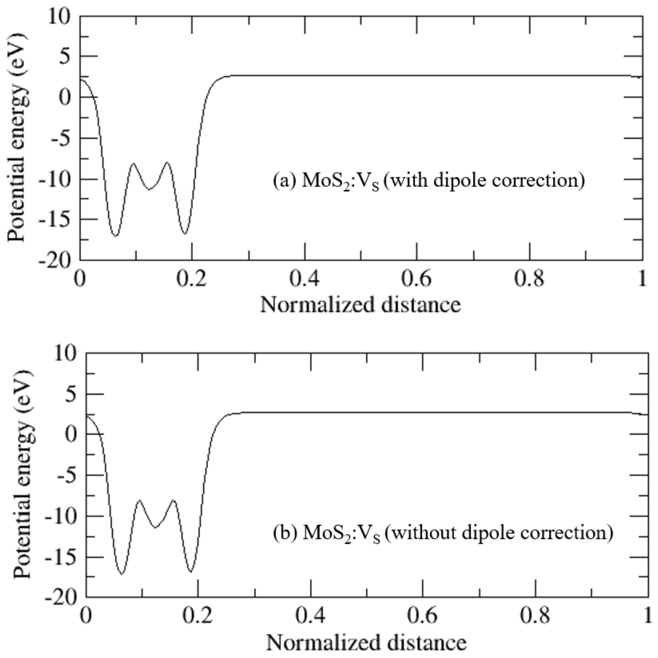

Supplementary Figure 13. DFT results on dipole correction. Plane-averaged local Hartree potentials for single sulfur vacancies in $mMoS_2$ films with (**a**) and without (**b**) dipole corrections.

Supplementary Table

			A (eV)	*A* (nm)	*B* (eV)	*B* (nm)	***A*-*B* (meV)**
**$mMoSe_2$**	On quartz	80 K	1.632	759.6	1.841	673.7	**209**
	On quartz	300 K	1.574	787.8	1.773	699.3	**199**
	On *h*-BN	80 K	1.628	761.6	1.823	680.2	**195**
	Sandwiched	80 K	1.631	760.3	1.832	677.0	**201**
	Sandwiched	300 K	1.570	789.9	1.769	701.1	**199**
**$mWSe_2$**	On quartz	80 K	1.675	740.4	2.111	587.3	**436**
	On quartz	300 K	1.623	764.1	2.035	609.2	**412**
	On *h*-BN	80 K	1.712	724.2	2.128	582.7	**416**
	Sandwiched	80 K	1.742	712.0	2.169	571.8	**427**
	Sandwiched	300 K	1.681	737.8	2.094	592.2	**413**
**$mMoS_2$**	On *h*-BN	80 K	1.927	643.5	2.078	596.8	**151**
**mWS_2**	On *h*-BN	80 K	2.041	607.4	2.417	513.1	**376**

Supplementary Table 1. Exciton peaks of SC-mTMD films from optical transmittance measurements. *A*- and *B*-exciton peak positions of $mMoSe_2$, $mWSe_2$, $mMoS_2$, and mWS_2 from transmittance spectra in different dielectric environments: monolayer films on quartz substrates (on quartz), monolayers on *h*-BN and quartz (on *h*-BN), and encapsulated monolayers with *h*-BN flakes and placed on quartz substrate (sandwiched). Both *A*- and *B*-exciton peaks are more susceptible to nearby dielectric environments and temperature variations than the rather unresponsive *A*–*B* exciton spacings, whose values are estimated to be 200.6 ± 5.2 meV for $mMoSe_2$ and 420.8 ± 10.4 meV for $mWSe_2$ films. Uncertainty levels are further improved to 198.3 ± 3.1 meV for $mMoSe_2$ and 418.7 ± 7.4 meV for $mWSe_2$ when considering the case with high-quality non-interacting *h*-BN layers. We are thus able to assign with full confidence the SOC-induced valence band splittings of the four SC-mTMD films in the graphite–*h*-BN–graphene planar tunnel devices with an uncertainty level of ± 0.01 eV (Supplementary Figure 6), and later utilize them for analyzing the electronic structures of the semiconducting films with data from electron tunneling spectroscopy measurements.
